# Hospital and Institutional News

**Published:** 1912-11-09

**Authors:** 


### HOSPITAL AND INSTITUTIONAL NEWS.

### THE ILLNESS OF THE TSAR'S SON.

Amid the distracting vicissitudes of the Home Rule Bill and the war in the Balkans, the British public has so far paid but scant attention to the details of the indisposition from which the Tsar's heir is now recovering. But in Russia it is easy to comprehend that very serious alarm has been aroused by the recent pronouncement of the Court medical advisers to the effect that the boy is a hæmophilic—in other words a "bleeder." This extraordinary malady has cropped up at intervals since the early Middle Ages among scions of European Royal families. In popular language, the victims of this constitutional tendency to bleed excessively after comparatively slight injuries have been said to have "only one skin instead of three." The truth is, of course, that hæmophilics have just as many skins as anyone else (to wit, one); but that their blood lacks to a greater or less degree that power of coagulation or clotting which in normal persons leads to natural arrest of hæmorrhage after any except very serious injuries to large blood-vessels. At the present time it would seem that hæmophilia is more prevalent than ever among princes. The late Duke of Albany suffered from it, and eventually died of it. The second son of the King and Queen of Spain is said to be afflicted by it, and to be incurably deaf in consequence of hæmorrhage into the internal ear. Rumours are also current that the Hohenzollerns are not exempt from it, and that some of Queen Mary's relations are hæmophilic to a slight degree. The disease hardly ever occurs in females; but it is handed down almost exclusively through the female line. That is to say, the children of a male hæmophilic are not likely to suffer from the disease, whereas the male offspring of a female whose brothers are "bleeders" are eligible for the display of hæmophilia. In view of the youth of the Tsar's son, and the serious illness through which he has passed, the constitutional delicacy from which he appears to suffer is bound to occasion the utmost concern for his future among the whole Russian people, and the greatest sympathy throughout Europe.

### A WEST LONDON HOSPITAL APPOINTMENT.

The governing board of the West London Hospital have appointed Mr. H. S. Souttar, M.B., F.R.C.S., to the vacant assistant surgeoncy caused by the death of Mr. Bidwell, and the subsequent promotion to his place on the senior staff. It is being stated in surgical circles that the medical committee sent up a unanimous recommendation in favour of another candidate; but that the lay board overrode it and selected Mr. Souttar, who is surgical registrar at the London Hospital. If this is true the governors have followed the precedent set over the last vacancy for a gynæcologist at the West London, the peculiar circumstances of which we commented on at the time (The Hospital, December 17 and 31, 1910). We do not say or imply that in no case should a lay board go behind the recommendation of the medical committee in making appointments to a hospital staff. But we certainly do believe that only exceptional circumstances can justify such treatment. What has the medical staff of the West London Hospital done to merit the persistent way in which their endeavours to select suitable officers for the hospital are ignored? In submitting tamely to be overruled last time the medical staff, it is being said, were practically inviting a repetition of the snub. But the real point at issue is the maintenance of a supply of strong candidates for each vacancy, in order that a wide choice may be secured. If the impression gains ground that the goodwill of members of the lay board is more important to successful candidature than the recommendation of the medical staff, we fear that there will be a diminution in the supply of the best candidates, and this will in the long run prove a most unfortunate thing for the hospital.

### THE SALARIES OF POOR-LAW ASSISTANT MEDICAL OFFICERS.

Dr. H. W. Bruce, medical superintendent at Southwark Infirmary, East Dulwich Grove, in a report to the Board of Guardians, who have been discussing the qualifications of a third assistant medical officer, pointed out that the gentleman appointed was fully qualified and registered according to law, but that he had had no experience beyond that of a student. That, however, he reminded the Board, had been invariably the case with the infirmary assistant medical officers when they commenced duty. Many times it had been most difficult to obtain even one suitable candidate at once; although the appointment was advertised six times, only one fair application was received. The junior assistants were always men of no experience of institutions under the Poor Law, and they rarely stayed long enough to be really well up in the work. It was entirely a matter of salary. Dr. Bruce pointed out that the salary of the medical superintendent was only £450 a year, whilst those of the children's hospitals of the Metropolitan Asylums Board were £700, and in the fever hospitals £600 with students' fees. In Southwark the senior assistant received £150 and the junior £120; in the children's hospitals their salaries were £250 and £150 respectively; and in the fever hospitals £300 and £180 respectively. The Board decided to ascertain the salaries paid by other London Unions to their medical staffs. All this is only one side of the main issue which was explained in our columns so fully by Dr. C. T. Parsons three weeks ago. As the Southwark Guardians must be beginning to understand, unless the Poor-Law indoor medical service be made much more attractive, it will be soon impossible to obtain not merely suitable candidates for the junior medical posts, but any candidates at all. Such is the Nemesis that at last attends an underpaid service with no reasonable prospects of promotion.

### A MEDICAL SUPERINTENDENT LEAVES FOR THE FRONT.

Among the latest departures for the Balkans is Mr. R. D. O'Leary, of the Seamen's Hospital Society. Mr. O'Leary has been medical superintendent of the "Dreadnought" Hospital, Greenwich, for four years, and he goes out under the auspices of the Red Cross Society. On Wednesday last week, October 30, Mr. O'Leary was entertained at Frascati's by the staff and his colleagues at the "Dreadnought."

### THE PROPOSED NEW UNITS AT THE ROYAL FREE HOSPITAL.

The best comment to the letter from Lord Sandwich, which we publish this week on behalf of the proposed new out-patient department at the Royal Free Hospital, is to give a brief summary of the principal changes in contemplation. These are very comprehensive. Indeed, it is far more than a mere out-patient unit that it is hoped to build on the acquired site. The buildings, roughly speaking, will form an L shape, the long arm being devoted to the out-patient department, round the central hall of which, on the ground floor, the dispensary, almoners', surgeons', and physicians' rooms will be grouped. The ground floor of the short arm will contain the casualty block with its central hall, two sides of which will be flanked by women's and men's rooms respectively, while the third, opposite to that from which egress from the out-patient department is obtained, will be occupied by the theatre. Above this ground floor are two storeys. That immediately over the out-patient department will provide for the dental, *x*-ray, electrical, and massage departments, and for the students' quarters on one side, with those of the nurses on the other; while the first storey of the casualty block will contain the rooms of the resident domestic staff. The second floor, with the exception of further nurses' bedrooms connected by a private staircase with the suite below, comprises a complete maternity department. This unit is composed of a "Maternity House," the residential quarters of the obstetric staff, and of two wards, one of eight beds and a labour ward of three beds, together with a theatre. Ashley and Newman are the architects. Even so terse a description as this makes it plain how comprehensive is the scheme of extension proposed. The £50,000 asked for is thus in practice to be devoted not to one purpose, but to three: an out-patient and casualty block with their allied sections; a complete gynæcological unit; numerous nurses' bedrooms, to say nothing of the ward-maids' quarters. We emphasise this not with a desire to criticise the grouping of these departments, but to explain that £50,000 is asked not for a new out-patient department only, but for a far more ambitious scheme.

### THE PROPRIETARY MEDICINES INQUIRY.

Further medical evidence respecting the danger of the unchecked distribution of secret remedies was furnished by the Select Committee on Proprietary Medicines at the last sitting, when Professor W. E. Dixon, of Cambridge, appeared as a witness. Professor Dixon made special reference to the sale of headache powders consisting of acetanilide, a drug which, he said, was extremely dangerous. With regard to cough mixtures he pointed out that no attempt was made by owners of such remedies to distinguish between the different kinds of cough —the "useful" cough of an old man whose chest is full of fluid which is removed by the cough, and the "useless" cough such as that which occurs in the early stage of consumption, and ought to be stopped. Another matter to which the witness drew attention was the multiplication of trade names for the same substance; he expressed the opinion that this was objectionable, and suggested that one chemical compound should have one name only. This question has been discussed in The Hospital in articles in which it has been shown that chemists have to keep in stock half a dozen or more bottles of the same drug under a variety of fancy names. Professor Dixon said that he believed it would be possible to check the sale of remedies for which fraudulent claims are made by a stricter enforcement of the Merchandise Marks Act. At the close of the sitting the Chairman stated that the medical evidence before the Committee was, for the time being, at an end.

### THE CITY MEDICAL OFFICERSHIP.

The published list of candidates recalls that the Corporation are about to appoint a Medical Officer of Health for the City of London in succession to Dr. Collingridge, whose retirement is now taking effect. The salary will be £1,000 a year, and the gentleman appointed must devote all his time to the work. The appointment dates from Lady Day next, and candidates will be selected at a meeting of the Sanitary Committee on December 12. The final choice rests with the Court of Common Council. Prior to his appointment Dr. Collingridge, with whom we published an interview when his retirement was announced, was Medical Officer to the Port of London.

### PROTECTING THE PRACTITIONER.

The latest annual report just issued of the London and Counties Medical Protection Society contains interesting reading, especially with regard to law cases in which members have been involved. A perusal of the details of such cases forcibly presses home the fact that there are many unsuspected dangers environing the young practitioner. As an example of the style of action sometimes brought, let the following suffice: Two members in partnership, who were the medical officers to a Board of Guardians, were sued by a woman for damages in respect of a libel contained in a certificate signed by the members and sent to the relieving officer. Counsel was briefed by the Society on behalf of its members, a verdict was given in their favour, and judgment entered accordingly. Of course, no costs were recovered, and the Society, therefore, has had to pay its share of the costs. Notwithstanding this verdict against her, the woman in question has commenced another action against the two members, claiming damages for libel contained in certain repeated certificates given to the relieving officer. Many questions concerning alleged negligence and unskilfulness, libel and slander, ethical questions and professional secrecy, contracts, and a vast number of other difficult matters were satisfactorily settled by the Society last year. The Association ought to increase its sphere of usefulness considerably when the medical benefits under the Insurance Act come into operation. Indeed, under the new conditions it will doubtless have its hands pretty full in settling disputes and supervising agreements.

### A HOSPITAL FIRE BRIGADE'S GENEROUS ACTION.

It is not often that a hospital fire brigade has the opportunity of forestalling the public fire-engines, but that honour must be paid to the porters and night staff of the Queen's Hospital for Children, Bethnal Green, which has twice previously exhibited similar public spirit. A house two doors from the hospital building recently caught fire, and the fact was discovered by the night porter and night stoker. They promptly roused the other porters, and the hospital fire hose from two hydrants in the hospital was quickly got out, and two jets of water were thrown upon the flames till the arrival of the fire brigade was able to finish the matter. This hospital has a fire drill every month, and, as stated, has three times in recent years proved its readiness to act in emergencies. On one occasion the danger zone was a wood-turner's premises quite close to a timber yard, the dangers attendant on which we discussed only recently.

### DOUBLE CONSCIOUSNESS AS AN EXCUSE FOR CRIME.

Can a man steal a motor-car, alter the number under which it was registered, drive it away from the owner's premises, and be not responsible for his actions? This was the question which a jury at Derby Assizes had to decide last Saturday. The defence, which made such a question possible, was that the accused at the time of his actions, which were admitted by his counsel, was "in a condition known to medical men as automatism." Many cases of a similar kind were cited by Dr. Parry Jones, of Derby, for the defence. But Mr. Justice Scrutton waived them aside, stating that he had an uncomfortable feeling that if the prisoner had not been so well dressed, but had appeared in corduroys, and if that defence had not been put forward by eminent King's Counsel and supported by specialists, but had been spoken from the dock, the jury would have thought little of it. So far as he knew, it was the first occasion in England when the question of double consciousness had been raised in the Courts. The jury found the prisoner guilty, and a sentence of four months' imprisonment in the second division was passed.

### STEVENS *v.* BRITISH MEDICAL ASSOCIATION.

After a trial lasting nine days, the jury disagreed in this important libel action and were discharged without rendering a verdict. It has not been disclosed whether they were fairly evenly divided, or whether one side or the other came near to gaining the day; but it is easy to understand and to sympathise with the doubts and hesitations which a layman might naturally feel when called upon to adjudicate such a complicated and technical business. The average juryman can well be excused for failing to differentiate between the professional standing and attainments of the medical men who appeared for the complainant and of those who testified for the defence. Nor can he be expected to appreciate the fact that any man who asserts that the lung of a patient suffering from silicosis will bounce like a stone when dropped on to a hard surface is a man whose evidence is worthless. The attainment of wealth by advertising and selling secret remedies is so extraordinarily easy in this country that the wonder is more people have not adopted that means of livelihood. In particular, members of the medical profession would hold such a great advantage over the laymen who pursue this miserable traffic that it says a great deal for the general standard of professional morals that none of them has put his hands to it. It has been stated that another action will be brought by the same complainant against the British Medical Association. Doubtless each side will have profited by the experience of the late drawn battle; but it certainly seemed from the published reports as if the case for the defence was presented with less skill than that for the prosecution.

### THE NEW LABORATORIES AT CHARING CROSS.

By a very happy arrangement the delivery of the Huxley Lecture at Charing Cross Hospital by Professor Flexner was made to coincide in date with the ceremony of formally handing over the new laboratories for sanitary science and bacteriology to their new possessors. These laboratories, as we have already mentioned in these columns, have been constructed in the Medical School of Charing Cross Hospital, and form a series of which not only the University of London, but any university may be proud. On October 31 the large attendance assembled to welcome Professor Flexner was afforded an opportunity of viewing the very extensive structural and other alterations in the college, and of assisting at the ceremony with which the laboratories were declared open and handed to the care of the public health staff of King's College. This ceremony took place in the old anatomical theatre and was performed by Professor Flexner himself, who improved the occasion by delivering an address on the practical importance to the community of further opportunity for research on the prevention of infectious disease. He congratulated London on the possession of this fine new unit, which would be devoted to the discovery of what was not known and the teaching of what was at present known. Professor Flexner, in referring to the splendid endowments of the Rockefeller and Carnegie Institutes in the United States, expressed the hope that one day some equally munificent benefactor would do similarly profitable work for the Old Country. Mr. W. F. D. Smith accepted the laboratories on behalf of King's College and paid a generous tribute to the energy and painstaking enthusiasm of the Dean of the Medical School of Charing Cross Hospital, Dr. William Hunter.

### THE FACTORY WIFE.

According to Dr. King Brown, Medical Officer of Health for Bermondsey, the increasing number of girls who enter factories has produced indirectly serious dangers to public health. While candidly admitting that legislation has done what it can to safeguard the health of the factory worker, Dr. King remarked that on leaving school the modern girl, instead of entering domestic service, which it was fashionable to despise, looked out for employment in factories. At eighteen or nineteen, he proceeded, they thought of getting married, and "the result is extremely disastrous from every point of view." The young wife's want of experience in the elements of household management, Dr. King affirmed, not merely in many instances wrecked the happiness of the marriage, but manifested itself with disastrous result when she became a mother, as her children were in charge of an ignoramus who was the cause of ill-health or even literally the death of them. The double evil was that unemployment among men was being increased by the smaller paid women workers, who at the same time were entering upon work which Nature had never intended them to undertake. More and more as highly controversial measures, intended to affect the public health of the community to its general advantage, are introduced into a heterogeneous House of Commons does the scientist grow suspicious of invoking legislation. The problem here is clearly an economic one, and the institutional worker may thank providence that its solution lies outside the immediate purview of his responsibilities.

### THE RED CRESCENT MOVEMENT IN EGYPT.

Modern wars, between first-rate Powers at any rate, are more quickly decided as a rule than the wars of history, except where the country leads to a wearisome series of indecisive guerilla engagements; therefore, it is not improbable that the countries which have been relatively late in the day to organise their Red Cross units may find that the hour when these were required has passed before the units had time to arrive on the field. According to the *Times* correspondent, the Red Crescent movement in Egypt was making great progress ten days ago, and Lord Kitchener's name on the list of subscribers is advertised as having proved a very popular incentive. The first medical mission equipped by the Red Crescent Society in Egypt was ready to start last week, but at the time of writing it is doubtful in what condition it will find the military organisation of the Turks, which, according to all uncensored news, has been thrown into general confusion since the battle of Kirk Kilisse. Thus, while the Turkish losses may seem to invite in a special degree the services of the Red Crescent Societies of Europe, their effect on Turkish administration appears hardly to warrant preparedness and smooth working for them when they arrive. How much co-operation and system is required to make the Red Cross units effective, especially when their members are of different nationality to that of the troops engaged, will have become apparent inferentially from the detailed account of "Medical Aid for Soldiers in the Field," which, with an illustrative diagram, appeared in The Hospital last week.

### A NARROW ESCAPE AT GLASGOW.

A curious condition has been disclosed during the demolition of the oldest portion of the Glasgow Royal Infirmary, the block facing Cathedral Square. Faults in construction and defects in the materials employed have been revealed, according to the *Glasgow Medical Journal*, of such a serious nature as to show that the occupancy of the building has been a grave danger to the inmates. The block was designed by the celebrated Adam brothers, of Adelphi fame, and was erected between 1792 and 1794. It has always been greatly admired as a very fine example of the art of those designers; but the building appears to have reached an advanced state of decay. All the centre portion, which looks like solid masonry, was really a skeleton; and it had actually to be shored up to prevent its utter collapse. The pediment face was almost all cement, and crumbled away in the workmen's hands. The timbers which alone held up the structure had reached such an advanced state of decay that the master of the works reports that a serious disaster might have occurred at any time. It would be interesting to know whether the brothers Adam had anything to do with the actual building operations, or whether a local builder erected the house from their plans. At any rate, it is fortunate that the rebuilding scheme should have been adopted just in the nick of time; but the revelation that jerry-builders flourished in the eighteenth century is distinctly disagreeable and alarming.

### THE HOSPITAL PROBLEM AT DONCASTER.

The position of hospital affairs at Doncaster presents much interest at the present time. The Doncaster Infirmary, which added fifteen beds to its accommodation in the summer of 1911, has to admit that it is over £1,000 down on the year's work, a sum, that is to say, which roughly represents the cost of maintenance of this addition. The medical staff is strongly anxious for a new infirmary of 100 beds to be built, which would cost £30,000, and for which a site has been actually obtained. But the new infirmary, Canon Sandford pointed out, would cost £2,000 a year more than the present institution, so that, apart from the initial expense of building, £3,000 a year would have to be raised to keep the new infirmary solvent and efficient. At this juncture the medical staff, as represented by Dr. A. C. Wilson, boldly ask for £5,000 from each of the new collieries round Doncaster, so that the new infirmary may be proceeded with. Is there any alternative to the raising of the annual income? It has been suggested from time to time, and not only in connection with the colliery population of Doncaster, that cottage hospitals for pit patients should be built by colliery proprietors, which would serve the double purpose of attending to accidents on the spot, and of relieving in some degree the existing pressure on the infirmary's accommodation. Canon Sandford, however, fears that such cottage hospitals would have the effect of withdrawing some, if not all, of the workmen's support, on which the Doncaster Infirmary at present depends so largely. We do not believe that this would necessarily result, and should like to see an inquiry made in other colliery districts as to the effect on the general hospital of the erection of such accident-treating institutions. It is, of course, hardly the time to commend their inception if an appeal to the colliery proprietors to finance the new building scheme is to be pressed at once.

### THE KING EDWARD MEMORIAL AT WINDSOR.

Few of the many hospital memorials which have been raised to the memory of Edward VII. can have had a more successful termination than that achieved in the Royal borough. For the Chairman of King Edward VII. Hospital, Windsor, has received from Prince Christian a cheque for £1,557, with a letter in the following gratifying terms: "Our memorial fund, thanks to the generous response to our appeal, is, after paying off a large debt and raising a statue to an illustrious founder, still large enough to enable me to hand you to-day a balance of over £1,500." Suffice it to say that £11,017 was raised for this memorial, which is an example of what was proportionally possible everywhere that sufficient energy was displayed. Sir James Gildea, who is engaged in the difficult task of collecting particulars of the memorials to King Edward, by command of Queen Alexandra—whom, therefore, every hospital secretary will have the honour of assisting by sending particulars of every hospital memorial in his district—can have received few more satisfactory instances of completed schemes than this at Windsor.

### THE CLAIMS ON THE PUBLIC OF TROPICAL RESEARCH.

Certain prominent members of the City Sub-Committee for the Rubber Trade have issued a special appeal for further contributions to the fund of £100,000, towards which a Lord Mayor's Fund for the extension of the London School of Tropical Medicine was started in February. They point out that business quite as much as humanitarian grounds support the appeal, since the profits from the rubber trade would clearly increase if the health conditions on the plantations could be improved. This fact, indeed, has been recognised by the rubber companies, which have so far subscribed £44,000, or approximately half of the amount desired. The appeal certainly should attract the interest of those numerous individuals who survived with a substantial balance on the right side the rubber boom, which must be still within the living memory of even the most forgetful shareholder. The London School of Tropical Medicine is deservedly fortunate in being able to appeal to very diverse sensibilities; it comes home to those for whom the phrase "think Imperially" was a genuine inspiration; to others who realise that one of the most onerous responsibilities of Empire is the sanification of districts which, like Panama, can be made of world-wide importance provided that tropical disease is banished from them; and now to those who desire that the profits of the rubber industry may be further increased. A cause so good, however, needs no lengthy appeal to excite generosity, and the public may be trusted, we believe, to assist with its money the latest rival in the race for their affections of tuberculosis and cancer research.

### AN ADVOCATE OF THE CIRCULAR WARD.

The past ten years have seen a perfect host of fashions in hospital construction and in the ideas which various surgeons in Germany and at home have insisted on importing into the designing of theatres, ward units, and pavilions. The tendency now is towards a greater simplicity in every department, and it is beginning to be felt that an imposing administrative block, which in Germany at least threatened to absorb enormous sums of money with no adequate gain to the institution, should give place to much less ambitious and far more useful elevations. So, too, with the modern theatre. The tiled palaces not long ago in vogue have become now a stereotyped phrase of the lay newspapers, and many are found to say that the comparative perfection of an aseptic technique has made elaboration in the theatre, even including the now classic round corners, a work of supererogation in hospital construction; the fashion of the circular ward is now a fashion of the past. Probably it never attained the popularity of the tiled theatre, or the craze for an enormous number of beds, but one of its advocates has passed away by the death of Dr. Frederick Bagshawe. It was in the early seventies that he became attached to the medical staff of the Hastings and East Sussex Hospital, and there he took very great interest in its rebuilding, which, as many readers will need no reminding, was designed on the circular ward plan. Though this type is found in new designs for individual wards in some institutions, it has generally been abandoned on the ground of administrative inconvenience, and, with its polygonal modification, is now regarded as a "freak." Nevertheless, it has had important advocates, and Dr. Bagshawe deserves to be remembered for his connection with the movement that led to this design being adopted at Hastings.

### THE COMPLETED MEMORIAL AT WOLVERHAMPTON.

On Wednesday, the 13th instant, the new King Edward Memorial Wing of the Wolverhampton and Staffordshire General Hospital will be opened by Lord Dartmouth. The Hospital Saturday Special Committee, and in particular Mr. W. H. Harper, the assistant secretary of the hospital, have been much congratulated on their efforts, which have been ably assisted by the workmen's contributions. Mr. W. Smith will hand over the money to Lord Dartmouth, who is the hospital's president. The raising of the money for this new wing has involved a considerable amount of unostentatious work on the part of the committee and working men, who deserve credit for the way in which they have come forward.

### STUFFINESS OR DRAUGHTS?

There is a good deal of truth in Dr. Robert T. Edwards' explanation of the sleepiness that has sometimes been observed in Church congregations at service time. Speaking as Medical Officer of Health for Merionethshire, he criticises severely the lack of proper ventilation in churches generally, and urges that among the many committees to which are entrusted Church affairs, one should be held responsible for securing a pure atmosphere in the church itself. Such a duty properly belongs to the churchwardens, who should give instructions to the verger in this respect, but everyone knows that for some reason or other most churches are places of stuffiness and draughts. The latter defect is so troublesome that congregations fly to the former by way of false refuge, but we are glad to find a medical officer of health drawing attention to the fact that because a church is used for religious purposes it should not disdain its duty to worshippers in the humbler sphere of ventilation. It is curious to note that Dr. Edwards' criticism arose out of his contention that the Churches should not be afraid to denounce the owners of slum property, a suggestion which most people will agree is less likely to secure general assent; with his former criticism we are in complete agreement, and ecclesiastical architects everywhere, in the interests of public health, might well be canvassed on this question: "Why in most churches does there seem no alternative between stuffiness or draughts?"

### A NEW PAPER ON MEDICINE AND POLITICS.

Till Professor Benjamin Moore by pen and platform became an advocate of a State Medical Service, the propaganda in its favour was mainly individual and interrupted. Now the State Medical Service Association has struggled to its feet and claims 135 medical men among its members. Its first general meeting has now been held, and an earnest of future energy is the announcement that a new weekly medical journal is to be expected shortly. The Association's aims have been summarised in what must now be a well-known list of thirteen reasons in favour of the State organisation of medicine, which include, by the way, the nationalisation of hospitals. Under a Board of Health, controlled by a Minister of Public Health, with Cabinet rank, the whole Service would be administered, the hospitals, we are told, being used for consultative, operative, and therapeutic work, at the request of and in consultation with the patients and doctors. The secretary of the new Association is Mr. Charles A. Parker, M.R.C.S., and Dr. G. A. Heron is chairman of the executive committee.

### POOR-LAW DISPENSERS AND THEIR SALARIES.

In our issue of May 25, it will be remembered, we notified that the Local Government Board was to be memorialised by the Public Pharmacists' and Dispensers' Association with a view to having the present maximum salary of £180 per annum for Poor-Law dispensers increased. We are now glad to learn that the Local Government Board have replied that in future recommendations for a higher maximum salary will be considered in relation to special duties or long service. Although no fixed scale has been established above the existing one, it is now possible for Boards of Guardians to recommend that their dispensers should have a rise in salary where the circumstances justify an increase.

### A NEW ISOLATION BLOCK AT CHARTRES.

The large hospital at Chartres—that Mecca of French tourists with its noble cathedral of unique and surpassing beauty—has recently been enriched by the addition of a very up-to-date isolation block. This pavilion, which was provided by the generosity of one of the leading citizens, is constructed on the completely isolated cubicle system. Each compartment has three walls of glass partitions, and contains a complete nursing and surgical equipment. There is on both the two floors a central corridor from which the compartments can be entered and observed. The fittings are modern and all constructed on approved aseptic lines. The pavilion, naturally, is isolated well away from the main blocks of the hospital—a simple matter considering the extent of the grounds, which are beautifully laid out, to the great advantage of the patients and staff, a fact that we think it well to emphasise in view of the possibilities and value of hospital gardening which are very remarkably illustrated in our articles on hospitals in Germany.

### THIS WEEK'S DRUG MARKET.

Business in the Drug market has been quieter than is usual at this time of the year. There have been several changes in price, but few of special importance. Higher prices have been paid for balsam copaiba. Balsam tolu is tending lower in value. Emetine is again dearer, owing to the increased demand for the alkaloid for use in the treatment of dysentery. Ergot of rye has advanced in price. Manna is dearer. Oil of cloves, oil of coriander, and oil of sandalwood are tending higher in value. The price of olive oil is steadily advancing. Lower prices are being asked for jalap. Orris root has again advanced in price owing to scarcity. Saffron is dearer, with prospects of a further advance. Sugar of milk tends towards lower quotations. There has been a further change in the position of opium and its derivatives, the values of all of which have advanced this week, with prospects of further advances.

### TO OUR READERS.

Contributions are specially invited from any of our readers to these columns. They should deal with topical subjects and news. They must be authenticated for the information of the Editor only. The minimum payment if published is 5s. There is no hard-and-fast rule as to space, but notes of about twenty lines in length are preferred.

